# *FSP1*, a novel KEAP1/NRF2 target gene regulating ferroptosis and radioresistance in lung cancers

**DOI:** 10.18632/oncotarget.28301

**Published:** 2022-10-19

**Authors:** Nsengiyumva Emmanuel, Hongen Li, Jing Chen, Yilei Zhang

**Affiliations:** ^1^The Institute of Molecular and Translational Medicine, Department of Biochemistry and Molecular Biology, School of Basic Medical Sciences, Xi’an Jiaotong University, Xi’an 710049, China; ^2^Department of Thoracic Surgery, Ruyang People’s Hospital, Luoyang 471200, China; ^3^Shaanxi Stem Cell Application Engineering Research Center, Shaanxi Jiuzhou Biomedical Science and Technology Group, Xi’an 710065, China; ^4^Department of Thoracic Surgery, The First Affiliated Hospital of Xi’an Jiaotong University, Xi’an 710061, China; ^*^These authors contributed equally to this work

**Keywords:** KEAP1/NRF2, lung cancer, ferroptosis, radioresistance, therapy

## Abstract

In the study of "A targetable CoQ-FSP1 axis drives ferroptosis- and radiation-resistance in KEAP1 inactive lung cancers" which was published earlier in Nature Communications, the authors have identified a novel KEAP1/NRF2 target gene, *FSP1*, and demonstrated that *FSP1* plays an essential role in NRF2-mediated ferroptosis resistance and radioresistance in KEAP1-deficient lung cancer cells. Currently, many NRF2 target genes have been found to participate in the regulation of ferroptosis, and exactly which one plays a dominant role seems unclear. This study proposes that *FSP1* is the key effector in NRF2-mediated ferroptosis resistance and radioresistance in KEAP-deficient lung cancer cells, as we discussed in the manuscript.

## INTRODUCTION

KEAP1-NRF2 regulatory pathway plays a vital role in the protection of cells against oxidative damage. Regulation of NRF2 by KEAP1 through cul3-keap1 ubiquitin E3 ligase complex has been implicated in various diseases including cancer, which must be considered for novel therapeutic development. KEAP1 mutation frequently occurs in non-small cell lung cancers like lung adenocarcinoma (LUAD) and lung squamous cell carcinoma (LUSC), which is significantly associated with aggressive tumor growth, resistance to available therapies, and poor prognosis [1, 2]. Mechanistically, KEAP1 mutation-induced NRF2 stabilization initiates the transcription of antioxidant response element (ARE)-containing genes, including those involved in iron metabolism (like FTH1, FTL, HMOX1, etc.) or GSH metabolism (SLC7A11, GCLC, GCLM, GSS, TXNRD1, etc.), which contributes to cell survival and tumor development under increased oxidative or metabolic stress conditions [3, 4]. However, whether there are any additional functional targets of NRF2 that mediate its antioxidative function and confer resistance of cancer cells to environmental stress remains elusive.

Ferroptosis is known as one type of regulated cell death specifically induced by lipid peroxides, which are generated in the free radical chain reaction between lipids and reactive hydrogens in the presence of ferrous iron. The findings that tumor suppressors such as p53 and BRCA1-associated protein 1 (BAP1) engage ferroptosis in tumor suppression establish ferroptosis as a natural barrier to tumor development, highlighting the great potential of targeting ferroptosis for cancer therapy [5, 6]. However, metabolic reprogramming or genetic alterations (such as oncogenic mutations) frequently lead to ferroptosis evasion, mainly through regulating ferroptosis defence systems like SLC7A11/GPX4, FSP1/CoQ, GCH1/BH4 and DHODH/CoQ [7]. Thus, a thorough understanding of the mechanistic regulation of ferroptosis would facilitate the exploration of therapeutic strategies for targeting ferroptosis in cancer. As a major antioxidative system within cells, NRF2 has been shown to prevent toxic accumulation of reactive oxygen species and suppress ferroptosis in response to oxidative stress through transcriptional regulation of SLC7A11, GPX4 and GCLC [8–10]. However, whether there are additional targets of NRF2 that contribute to its anti-oxidant function during tumor development remains an open question in the field.

Ferroptosis suppressor protein 1 (FSP1, previously known as AIFM2) has been identified as an novel GPX4-independent ferroptosis defence system since 2019. Modification of FSP1 by myristoylation leads its localization to the plasma membrane where it serves as an oxidoreductase to reduce coenzyme Q10 (CoQ), which functions as a natural lipophilic radical-trapping antioxidant to neutralize lipid peroxides [11, 12]. Moreover, FSP1 expression is highly correlated with ferroptosis-inducing agents (FINs, such as RSL3, ML162, ML210 and erastin) across a wide range of cancer cell lines, and potentially predicts ferroptosis sensitivity in lung cancer cells [12]. These findings highlight the potential of targeting FSP1 as a strategy to overcome ferroptosis resistance in cancer therapy. Thus, the mechanistic study of FSP1 expression absolutely raises more opportunities regarding FSP1-centered anti-cancer treatments. In a recent study by Pranavi Koppula et al. from The University of Texas MD Anderson Cancer Center, FSP1 was demonstrated as a novel target of NRF2 and to play a vital role in KEAP1/NRF2-mediated ferroptosis regulation [13], which reveals the important role of genetic regulation of FSP1 in cancer development.

After knockout of KEAP1 in lung cancer cells, the authors found upregulation of NRF2 and SLC7A11 expectedly, but the GPX4 level even decreased in these lung cancer cell lines. Surprisingly, deleting NRF2 in KEAP1-deficient cells restored mRNA and protein levels of GPX4, indicating a negative regulation of GPX4 by NRF2 in these cell lines [13]. These findings argue against the previous report of GPX4 as a transcriptional target upregulated by NRF2 [14], leaving how exactly GPX4 is regulated by NRF2 unsolved. Of note, KEAP1-knockout cells were more resistant to class 1 FINs (such as erastin) and class 2 FINs (such as RSL3 and ML162) but not to FIN56 (a class 3 FIN that depletes both GPX4 and CoQ), suggesting a role of KEAP1/NRF2 in the regulation of ferroptosis through employing CoQ pathway. Indeed, the authors performed a Gene Ontology analysis on the overexpressed genes in KEAP1-mutant tumors compared with KEAP1-WT ones from the Cancer Genome Atlas (TCGA) LUAD dataset and found that FSP1 is the most significantly upregulated one within the 12 genes involved in the CoQ metabolic process [13]. Further studies demonstrated that FSP1 expression level conferred ferroptosis resistance of KEAP1-mutant or -deficient lung cancer cells, since FSP1 inhibition by genetic knockout or pharmacological inhibitor re-sensitized KEAP1-deficient cells to ferroptosis, while FSP1 overexpression promoted ferroptosis resistance [13].

These findings suggest that FSP1 is a novel downstream effector of KEAP1/NRF2 in defending ferroptosis. Analyses of data from NRF2 chromatin immunoprecipitation coupled with high-throughput sequencing (ChIP-seq) indicated a strong association between NRF2 and FSP1 gene promoter in several cancer cell lines [13], guiding the authors to investigate whether FSP1 is a direct transcriptional target of NRF2. Next, two antioxidant response elements (AREs) which are well-established NRF2 binding motif were found in *FSP1* gene promoter regions. ChIP-qPCR assay was performed to verify the increased binding of NRF2 to the AREs of FSP1 upon KEAP1 knockout. Besides, NRF2 activator treatment or KEAP1 deficiency dramatically upregulated luciferase reporter activity of the FSP1 gene promoter, which was partially reduced by mutation of either ARE and nearly abolished by mutation of both AREs within the FSP1 promoter. Depletion of NRF2 erased the effects caused by KEAP1 deficiency, suggesting the indispensable role of NRF2 in the regulation of FSP1 expression and ferroptosis sensitivity [13].

As aforementioned above, KEAP1 mutation or deletion is frequently distributed in cancers. Thus, it’s necessary to determine the relevance of FSP1 to tumor development. Computational analyses of TCGA data revealed that FSP1 expression levels were significantly higher in multiple cancer types compared to their corresponding normal tissues. Experimentally, FSP1 deletion markedly repressed tumor growth of KEAP1-KO lung cancer cells in a xenograft model, suggesting that FSP1 is required for KEAP1-deficient lung tumor growth. Moreover, FSP1 suppressed the level of 4-HNE (a by-product during lipid peroxidation) in these tumors, suggesting FSP1-mediated ferroptosis resistance contributes to tumor growth of KEAP1-deficient cells [13].

KEAP1 mutant lung cancers are found radioresistant, which could be attributed to the ferroptosis resistance of these tumors [15]. Therefore, the authors examined the role of FSP1 in the regulation of radioresistance and found that NRF2-mediated FSP1 upregulation decreased lipid peroxidation level and promoted cell survival upon ionizing radiation (IR). FSP1 inhibition (using a FSP1 inhibitor iFSP1 or deleting FSP1 expression) largely promoted radiosensitization and IR-induced lipid peroxidation in KEAP1 deficient of mutant lung cancer cells [13], making FSP1 an ideal target for targeted treatment during radiotherapy. In addition, FSP1 is known to inhibit ferroptosis via regulating CoQ level, whether CoQ metabolism is involved in the radioresistance could be of great relevance. 4-CBA (which inhibits the key enzyme COQ2 involved in CoQ biosynthesis) treatment successfully restored radiotherapy-induced lipid peroxidation and caused tumor suppression in KEAP1-mutant lung cancer cells [13], highlighting the translational application of 4-CBA treatment in radiotherapy to overcome radioresistance in KEAP1-inactivated lung tumors.

**Figure 1:**
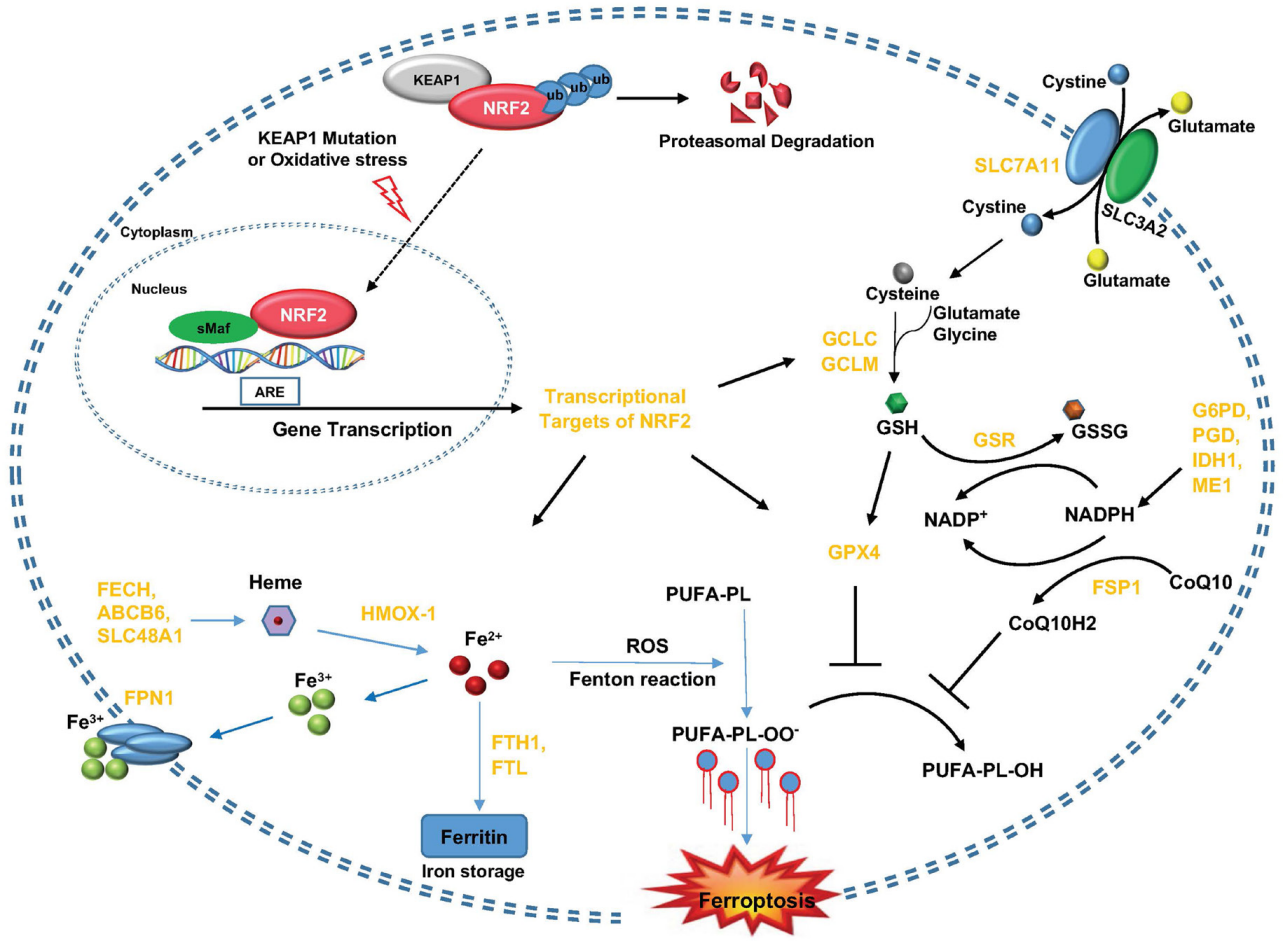
KEAP1-NRF2 axis and its molecular effectors in the regulation of ferroptosis. KEAP1 inactivation (like genomic mutation or conformational disruption under oxidative stress conditions) leads to the release of NRF2 translocation into the nucleus and enhances the transcription of target genes with ARE in their promoter regions. These NRF2 target genes could be divided into several groups based on their metabolic functions within cells, including cystine/GSH metabolism (SLC7A11, GCLC, GCLM, GSR, GPX4), CoQ metabolism (FSP1) and iron metabolism (FECH1, ABCB6, SLC48A1, HMOX-1, FPN1, FTH1 and FTL).

Currently, there are four major ferroptosis-defending systems, while NRF2 could directly control two of them, SLC7A11/GSH/GPX4 axis and CoQ/FSP1 axis. Besides, NRF2 also has transcriptional targets involved in iron metabolism (through FTH1, FTL, HMOX1, etc.) and NADPH regeneration (through G6PD, PGD, IDH1, ME1, etc.), which regulates ferroptosis sensitivity through modulating the levels of ferrous iron and NADPH. These findings seem to nominate NRF2 as a master regulator of ferroptosis, since all of these targets of NRF2 might, partially or completely, participate in the regulation of ferroptosis by KEAP1/NRF2. However, the regulation of NRF2 targets could be highly context-dependent. For example, GPX4 could be either upregulated or downregulated by NRF2 in different cancer cell lines [13]. Therefore, exactly which target(s) plays a major role in NRF2-mediated regulation of ferroptosis and cellular redox homeostasis should be exploited case by case (Figure 1). Here, Pranavi Koppula and her colleagues’ study indicates that pharmacological targeting of CoQ-FSP1 signaling to overcome KEAP1 deficiency-induced radioresistance could be a potentially effective therapeutic strategy in treating KEAP1 mutant lung cancers.
